# Meat quality traits and Blunt Meullenet-Owens Razor Shear characteristics of broiler breast fillets affected by woody breast condition and post-cooking meat temperature

**DOI:** 10.1016/j.psj.2021.101212

**Published:** 2021-04-21

**Authors:** Xiao Sun, Aline Giampietro-Ganeco, Ashleigh Mueller, Clay J. Maynard, Juan P. Caldas-Cueva, Casey M. Owens

**Affiliations:** ⁎ School of Biological Science and Food Engineering, Chuzhou University, Chuzhou, 239000, Anhui, China; † Department of Food Engineering, University of São Paulo - USP, Pirassununga, 13635-900, São Paulo, Brazil; ‡ Department of Poultry Science, University of Arkansas, Fayetteville, Arkansas 72701, USA

**Keywords:** woody breast, meat quality, cooking time, shear analysis, shear temperature

## Abstract

This study aimed to investigate meat quality attributes, cooking performance, and water properties of woody breast (**WB**). A total of 48 broiler breast fillets (7 wk, 3 h debone) of 24 normal (**NORM**) and 24 severe WB (**SEV**) were collected. Raw meat characteristics (L*, a*, b*, pH, compression force and energy and) along with the blunt blade of the Meullenet-Owens razor shear (**BMORS**) properties were determined. Cooking time and internal meat temperature were recorded for each fillet every 5 min on each fillet during cooking. Water/moisture properties and shear values of BMORS were determined at different meat temperatures (HOT [68°C], AMBIENT [22°C] and COLD [4°C]) after cooking. SEV fillets showed higher L*, a*, b*, pH, CF, CE, BMORS force, BMORS energy, and peak counts of BMORS values compared to NORM fillets in raw state (*P* < 0.05). Cooking time was shorter in SEV fillets than NORM fillets (*P* < 0.0001). Cook loss, total water loss, and moisture loss (HOT, AMBIENT) were greater in SEV fillets than NORM fillets (*P* < 0.01). PC-BMORS were greater in SEV fillets than NORM fillets (*P* < 0.05), and all BMORS shear values increased as post-cooking meat temperature decreased (*P* < 0.05). Positive correlations were observed between WB scores and raw meat characteristics and shear values. There were also significant relationships (*P* < 0.001) between WB scores and cooking performance measures except moisture loss for COLD treatment. BMORS force and energy were moderately correlated to total water loss, cook loss, and moisture loss (HOT) regardless of meat temperature (*P* < 0.05); however, PC-BMORS was only correlated to total water loss at COLD and moisture loss (HOT) at all meat temperatures (*P* < 0.05). These data corroborate the association of WB condition with impaired quality/texture characteristics in raw and cooked fillets; WB also had a significant impact on cooking time, cooking at a faster rate, along with water/moisture loss during and after thermal processing. Results demonstrate that the post-cooking meat temperature plays an important role in shear test values.

## INTRODUCTION

Woody breast (**WB**) is an emerging and challenging myopathy of broiler *Pectoralis major* muscle in the global poultry industry. WB is characterized by an abnormal hardness typically detected using subjective palpation and visual evaluations of ridge like bulge on the caudal region. Breast fillets affected by WB condition exhibit increasing breast weight, pH, fat and connective tissue content with lower levels of proteins (Sihvo et al., 2014; Mazzoni et al., 2015; Soglia et al., 2016b; Tijare et al., 2016; Petracci et al., 2019). The histology and muscle composition differences in WB result in meat quality defects which lead to impaired functional properties such as a poor water holding capacity (**WHC**) and lower acceptance scores in sensory attributes (Tasoniero et al., 2017; Cai et al., 2018; Bowker and Zhuang, 2019; Caldas-Cueva et al., 2020; Xing et al., 2020a,b; Caldas-Cueva et al., 2021b). In recent years, the increasing incidence rates of WB condition in the poultry industry have caused important economic losses, in millions of dollars, during primary and further processing operations (Kuttappan et al., 2016; Barbut, 2019; Petracci et al., 2019; Caldas-Cueva and Owens, 2020; Hanning et al., 2020). Besides the differences of physicochemical and texture properties in raw WB meat, moisture/water properties in WB could also be different compared to unaffected fillets. Previous studies (Soglia et al., 2016a, b; Pang et al., 2020a,b) reported that raw WB meat had greater moisture content compared to normal fillets and more moisture losses (drip loss) during short term cold storage (Sun et al., 2018). A study also reported greater shear force values for cooked meat samples tested at 20°C in comparison with those assessed at 70°C (Ledward and Lawrie, 1975). Meanwhile, Solo (2016) indicated better sensory results (higher scores of tenderness/juiciness) of breast fillets with WB condition serving in hot meat temperature compared to when served cold. Those results demonstrate the effect of WB condition on meat quality and water properties in raw meat state as well as the association of post-cooking/serving meat temperature with texture properties and sensory evaluations of cooked meat. However, the effects of water properties on cooking performance and the relationships between water properties, shear values, and WB condition are still not well understood. Therefore, the objectives in this study were to determine 1) effects of WB condition on meat quality traits in raw meat, cooking time and water/properties (cook loss, moisture loss and total water loss); and 2) effects of WB condition and post-cooking meat temperature on shear values in cooked meat.

## MATERIALS AND METHODS

### Sample Preparation

High-yield male broilers were processed at 7 wk of age under a commercial-style in-line processing system (Mehaffey et al., 2006) at University of Arkansas Pilot Processing Plant. A total of 48 butterfly breast fillets (deboned at 3 h postmortem) were collected and categorized into normal (**NORM**) and severe woody breast or WB (**SEV**) groups (n = 24/category, Tijare et al., 2016; Sun et al., 2018). After scoring, all butterfly breast fillets were split into left and right fillets; right fillets were used for all analysis (left fillets discarded) and were individually packed in zip-sealed plastic bag, placed on ice, and stored in a walk-in cooler at 4^◦^C for analysis.

### Color and pH Analysis

Color and pH of breast muscle were measured at approximately 24 h postmortem. Breast fillet color was recorded with a handheld Minolta colorimeter which was configured using SpectraMagic NX software (Minolta CM-400, Konica Minolta Sensing Americas Inc., Ramsey, NJ, USA), set with a 2-degree observer, decreasing surface reflectance, and illuminant of D_65_. Prior to obtaining color values, the colorimeter was calibrated to manufacturer recommendations utilizing the provided standard white calibration tile. Calibration values were entered according to the Y, x, and y calibration scheme (D_65_) and entered as 84.8, 0.3203, and 0.3378, respectively. Color values (L*, a*, b*) were measured 3 times (cranial, medial and caudal locations) on the dorsal surface (bone side, in contact with the *Pectoralis minor* muscle), then the average values of L*, a*, b* were recorded respectively. Muscle pH was measured in the cranial end of fillet (near the wing joint area) using a pH meter equipped with a combination spear tip electrode (Model 205, Testo instruments, West Chester, PA, USA).

### Compression and BMORS Texture Assessments in Raw Breast Fillets

**Figure 1:**
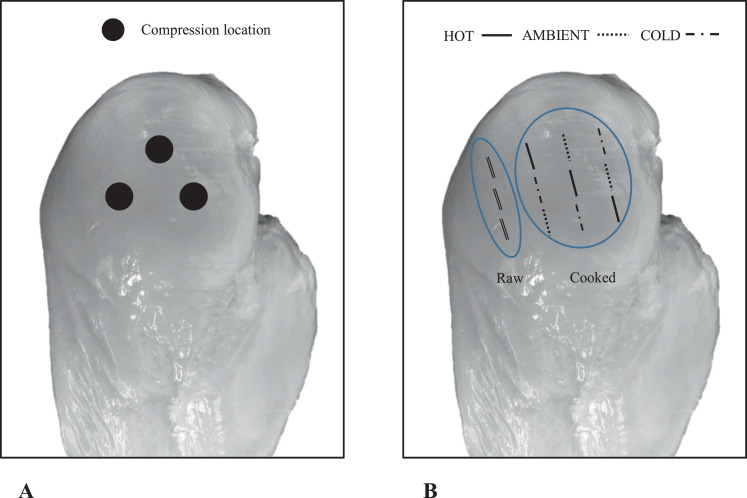
Compression and shearing locations of breast fillets. For each raw breast fillet, compression force and compression energy were conducted on 3 different locations in the cranial region of ventral side as shown in panel A. BMORS measurements in raw and/or cooked fillet were determined at 3 different post-cooking meat temperature treatments [(HOT (68°C), AMBIENT (22°C) and COLD (4°C)] and 3 different locations in the cranial region of each breast fillet as shown in panel B. Double solid black lines on the left part of the breast fillet represented shear measurements in the raw state. The other black lines on the right part of the breast fillet represented shear measurements in the cooked state assessed at 3 different post-cooking meat temperatures (HOT, AMBIENT, and COLD, respectively).

Compression analysis was carried out according to Sun et al. (2018) with slight modifications. Breast fillets were compressed to 30% of fillet height 3 times on cranial region (Figure 1A) using a 6 mm flat probe on a texture analyzer (Model TA.XT Plus, Texture Technologies Corp., Scarsdale, NY, USA), and the average compression force (**CF**) as well as compression energy (**CE**) were recorded. The trigger force was set at 5 g, load cell capacity of 5 kg, probe height set at 55 mm (higher than the thickest fillet sample), pre- and post-probe speeds were both 10 mm/s, and the test speed of the probe was 5 mm/s.

After compression analysis, same breast fillets were sheared perpendicularly to the muscle fibers with 3 shears per fillet on the cranial region (Figure 1B) using the blunt version of Meullenet-Owens razor shear (BMORS) on texture analyzer (Model TA.XT Plus, Texture Technologies Corp., Scarsdale, NY, USA). The TA.XT Plus Texture analyzer with a load cell capacity of 5 kg was set at crosshead speed of 5 mm/s along with a sample shear depth of 20 mm and a trigger force of 5 g. The results of BMORS shear readings of raw fillets were reported as average shear force (BMORSF, N), shear energy (BMORSE, n.mm) and peak counts of shear curves (PC-BMORS).

### Breast fillet Physical Measurements

Fillets were horizontally sliced to simulate portioning practices in industry to achieve approximately 25-30 mm in thickness. When fillets were in this range initially, the fillet was still sliced so that a cut edge would be present in all fillets. The ventral side of each fillet after portioning was used for further analysis and the dorsal portion was discarded. Breast weight along with cranial thickness (the thickest point in the cranial region of the breast fillet) of right-side breast fillets were recorded after portioning.

### Breast Fillet Cooking

After trimming, all portioned breast fillets were kept in a walk-in cooler 4°C and cooked at d 1 (24 h postmortem). Breast fillets were cooked on raised wire racks with the sliced side down in aluminum foil covered pans (4 per pan) with an air convection oven (Sams et al., 1990; Tijare et al., 2016). Eight breast fillets (4 NORM, 4 SEV) were cooked each time, and a total of 5 cooking replications (n=40) were carried out in this study. Internal fillet temperatures were recorded individually by inserting a wire into the fillet throughout the cooking process; temperature was monitored using a multi-channel Digi-sense scanning thermometer (Model 69200-00; Barnant Co., Barrington, IL, USA). Before cooking, temperature of all samples was recorded at time 0 min, and then every 5 min during cooking until the internal end-point temperature reached a minimum of 76°C (not greater than 78°C). Cooking time (min) of each breast fillet was also recorded.

### Cooking Performance

In this study, water/moisture properties (related to water holding capacity) were investigated during/after thermal processing of breast fillets. Breast muscle weight recorded at certain time/temperature point which were before and after cooking (76°C), cooled around 3 min with fillet temperature at 68°C (hot), cooled to ambient room temperature (22°C) and cooled to 4°C (overnight in refrigerator), respectively. Cook loss was calculated by the percentage of weight change before cooking and directly after cooking but prior to shearing. Three different moisture loss were determined right after cooking until sample cooled down to 4°C with the following description. Moisture loss of hot (76°C−68°C; ML-HOT) was calculated by the percentage weight change of fillet right after cooking and fillet temperature at 68°C; Moisture loss from hot to ambient room (68°C−22°C; ML-AMBIENT) was calculated by the percentage of weight change of fillet temperature at 68°C and 22°C, respectively. Moisture loss from room to cold (22°C−4°C; ML-COLD) was calculated by the percentage of weight change of fillet temperature at 22°C and 4°C, respectively. Then the total water loss of breast fillet was calculated by the percentage of weight change before cooking and fillet temperature at 4°C.

### BMORS Assessment in Cooked Meat at Varying Post-cooking Temperatures

Texture analysis of portioned breast fillet were conducted at 3 different post-cooking meat temperatures ranging from 68°C to 4°C and classified as HOT (68°C), AMBIENT (22°C) and COLD (4°C) treatments. In the present study, shear values were obtained using BMORS method with texture analyzer (Model TA.XT Plus, Texture Technologies Corp., Scarsdale, NY; Cavitt et al., 2005; Mehaffey et al., 2006; Lee et al., 2008). Briefly, fillets were sheared 3 times in their cranial region at each temperature treatment (HOT, AMBIENT, and COLD) as shown in Figure 1B. The results were averaged per fillet and reported as shear force (BMORSF, N), shear energy (BMORSE, N.mm) and peak counts of shear curves (PC-BMORS, Sun et al., 2016, Bowker and Zhuang, 2019).

### Statistical Analysis

Data from this study were analyzed using the GLM procedure in JMP (SAS Institute Inc., Cary, NC). Meat quality traits in raw breast fillets were analyzed by testing the main effect of WB condition (NORM, SEV). For cooking performance, cooking time and moisture/water properties of breast fillet (cook loss, moisture loss and total water loss) were analyzed by testing the main effect of WB category (NORM, SEV). For texture analysis of BMORS in cooked breast fillet, shear properties of BMORS (BMORSF, BMORSE and PC-BMORS) were analyzed by using repeated measures analysis (mixed model) in JMP to test the effects of WB category (NORM, SEV), post-cooking meat temperature of breast fillet (HOT, AMBIENT and COLD), and the interaction of WB category and post-cooking meat temperature. Means were separated by Tukey's Honestly Significant Difference test for multiple mean comparison or Student *t* test and the significance level was set at *P* < 0.05. Spearman's correlation coefficients (r_s_) between raw/cooked meat quality traits and woody breast scores were determined. The relationship between shear values of BMORS and moisture/water properties by post-cooking meat temperature were analyzed by calculating Pearson correlation coefficients (r). Additionally, correlations among all water/moisture properties were analyzed by calculating Pearson correlation coefficients (r).

## RESULTS

### Meat Quality Attributes in Raw Breast Fillet

Meat quality traits of raw breast fillets with WB condition in comparison with normal fillets are shown in Table 1. CIE L*a*b* color measurements on dorsal side of fillets and pH were different between WB categories where SEV fillets had greater CIE L*a*b* and pH values than NORM fillets (*P* < 0.05). Similarly, SEV fillets showed higher CF and CE values as well as BMORSF, BMORSE and PC-BMORS parameters when compared to NOR fillets (*P* < 0.05). Weight and thickness values were slightly (<15%), but significantly different between WB groups (P < 0.0001). The average values of breast weight and thickness were greater for SEV fillets compared to NORM fillets.

**Table 1:** Meat quality traits of raw breast fillets with woody breast (WB) condition and Spearman's correlation coefficients (r_s_) to WB scores.

	WB category^1^				
Parameter	NORM	SEV	SEM	*P* Value	Correlations to WB scores
Weight (g)^2^	211.71^b^	234.75^a^	4.35	0.0067	–
Thickness (mm)^2^	25.75^b^	29.44^a^	0.38	<0.0001	–
Color					
L*	55.58^b^	58.26^a^	0.47	0.0029	0.42⁎⁎
a*	3.24^b^	3.96^a^	0.15	0.0144	0.35⁎
b*	9.98^b^	11.28^a^	0.24	0.0051	0.40⁎⁎
pH	5.88^b^	6.07^a^	0.02	<0.0001	0.76⁎⁎⁎
Compression^3^					
CF (N)	3.88^b^	15.10^a^	1.05	<0.0001	0.78⁎⁎⁎
CE (N.mm)	12.77^b^	72.19^a^	5.56	<0.0001	0.78⁎⁎⁎
BMORS^4^(raw)					
BMORSF(N)	9.96^b^	12.77^a^	0.55	0.0091	0.37⁎⁎
BMORSE (N.mm)	97.99^b^	129.95^a^	5.65	0.0036	0.41⁎⁎
PC-BMORS	6.15^b^	7.36^a^	0.18	0.0004	0.49⁎⁎

1 NORM = normal, fillets were soft and flexible throughout; SEV= severe, fillets were extremely hard and rigid throughout with limited or flexibility from cranial to caudal region, all meat quality traits were conducted on breast fillets before trimming.

2 Portioned fillet.

3 Compression measurements: CF = compression force; CE = compression energy.

4 Blunt Meullenet-Owens Razor Shear (BMORS) features in raw meat. BMORSF=BMORS shear force (N); BMORSE=BMORS shear energy (N.mm); PC-CBMORS=peak counts of BMORS.

a-b Means within the same row followed by different superscript letters differ significantly (*P* < 0.05).

⁎⁎⁎ *P* < 0.001; ^⁎⁎^*P* < 0.01; *P < 0.05.

### Cooking Performance of Breast Fillet

Cooking performance (cooking time and water/moisture properties) of breast during/after cooking are shown in Table 2. Cooking time, cook loss, total water loss, moisture loss (HOT, AMBIENT) were different between WB categories (*P* < 0.001). Interestingly, the cooking time (to an internal temperature of 76°C) for SEV fillets (35.80 min) was shorter than that for NORM fillets (43.88 min, *P* < 0.0001). On the other hand, the cook loss of SEV fillets (21.71%) was greater (*P* = 0.0004) than NORM samples (15.27%). For moisture loss after cooking, SEV fillets presented higher (*P* < 0.0001) moisture loss of HOT (3.23% vs 4.99%) and AMBIENT (2.14% vs 4.42%) compared to NORM fillets respectively; however, COLD moisture loss of NORM was comparable to that of SEV fillets (*P* > 0.05). Similar to cook loss, SEV fillets were associated with greater total water loss in comparison with NORM fillets (29.55% vs 20.72%, respectively; *P* < 0.0001).

**Table 2:** Cooking performance of cooked breast fillets with woody breast (WB) condition and Spearman's correlation coefficients (r_s_) to WB scores.

	WB category^1^				
Parameter	NORM	SEV	SEM	*P* Value	Correlations to WB scores
Cooking time (min)	43.88^a^	35.80^b^	1.48	<0.0001	-0.53^⁎⁎⁎^
Cook loss (%)	15.27^b^	21.71^a^	0.96	0.0004	0.61^⁎⁎⁎^
Moisture loss (%)					
HOT^2^	3.23^b^	4.99^a^	0.20	<0.0001	0.63^⁎⁎⁎^
AMBIENT^3^	2.14^b^	4.42^a^	0.32	<0.0001	0.71^⁎⁎⁎^
COLD^4^	1.22	0.90	0.12	0.0568	-0.30
Total water loss (%)	20.72^b^	29.55^a^	0.94	<0.0001	0.73⁎⁎⁎

1 NORM = normal, fillets were soft and flexible throughout; SEV = severe, fillets were extremely hard and rigid throughout with limited or flexibility from cranial to caudal region.

2 HOT = moisture loss from 76°C to 68°C.

3 AMBIENT = moisture loss from 68°C to 22°C.

4 COLD = moisture loss from 22°C to 4°C.

a-b Means within the same row followed by different superscript letters differ significantly (*P* < 0.05).

⁎⁎⁎ *P* < 0.001.

**Figure 2:**
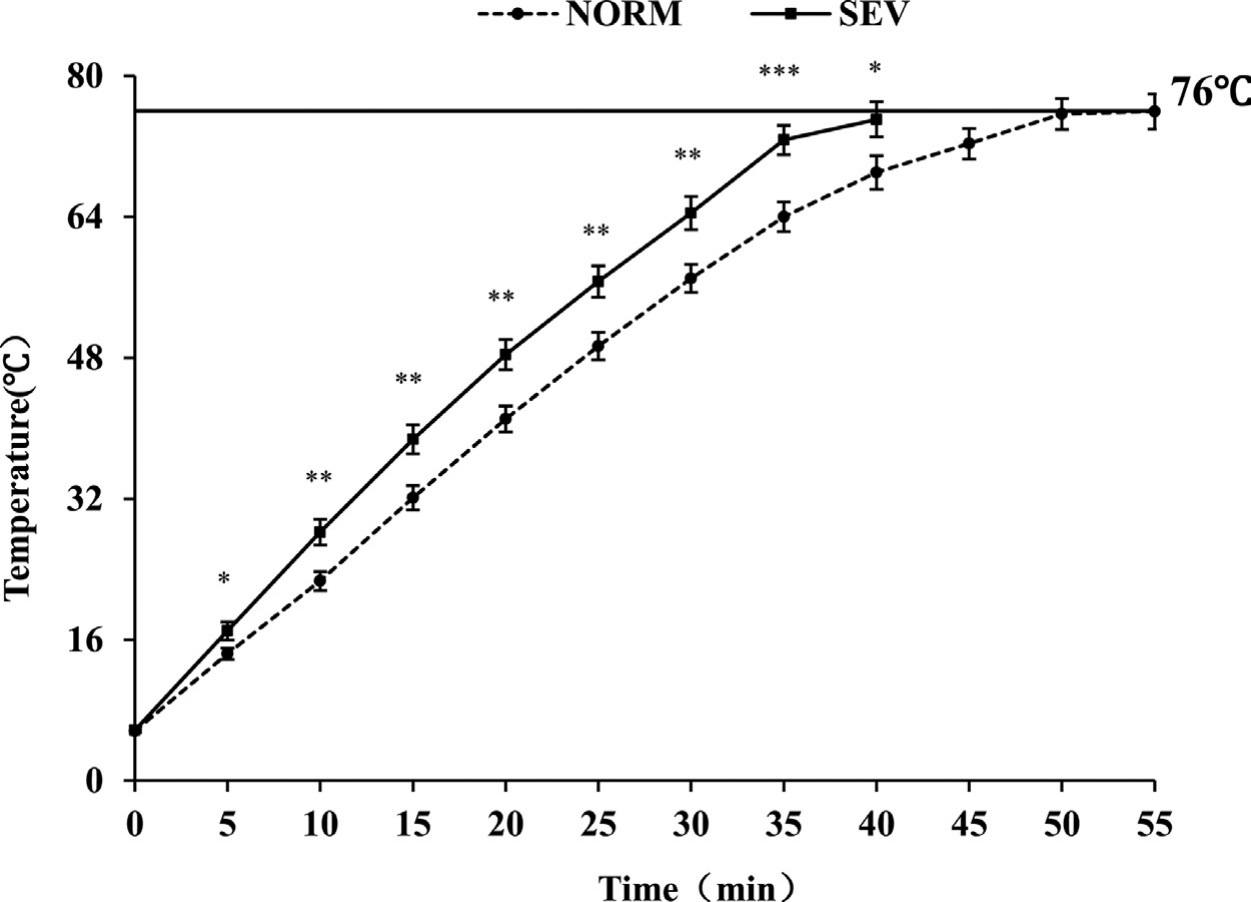
Meat temperature change of normal (NORM) and severe woody breast (SEV) fillets during cooking. For each time point, n = 20/mean (0−35 min), n = 16/mean (40 min), n=12 (only NORM 45−50 min), n = 4 (only NORM 55 min).

Meat temperature changes during cooking of NORM and SEV fillets are shown in Figure 2. Overall, NORM fillets required longer time to reach an internal meat temperature of 76°C compared to SEV fillets. At time 0 min, there was no difference (*P* > 0.05) between WB categories, but as cooking time increased (from 5 min to 40 min), SEV fillets had a higher internal meat temperature compared to NORM fillets on each temperature recording point (*P* < 0.05).

All fillets were cooked to a final core temperature of 76°C. ^⁎⁎⁎^means *P* < 0.0001, ^⁎⁎^means *P* < 0.01, *means *P* < 0.05, no asterisk means *P* > 0.05 between NORM and SEV fillets in each time period during cooking.

### Texture Analysis of BMORS in Cooked Breast Fillet with Different Post-cooking Meat Temperatures

Table 3 shows the effect of WB condition and post-cooking meat temperature on shear values of BMORS. The BMORSF and BMORSE values were similar between NORM and SEV while PC-BMORS was significantly higher in SEV fillets than NORM fillets (*P* < 0.0001). Significant differences were found in BMORSF, BMORSE and PC-BMORS among post-cooking meat temperatures (*P* < 0.0001). However, no significant differences were noted for interaction between WB category and post-cooking meat temperature factors (*P* > 0.05). Shear values of BMORS increased as meat temperature decreased (HOT < AMBIENT < COLD: *P* < 0.05). The greatest BMORSF, BMORSE and PC-BMORS values were observed in COLD intermediate values in AMBIENT, and the lowest values in HOT in both NORM and SEV fillets. An exception was noted in the BMORSF of NORM fillets sheared at AMBIENT or COLD temperature in which they did not differ (*P* > 0.05).

**Table 3:** Effect of woody breast (WB) condition and post-cooking meat temperature on shear values using Blunt Meullenet-Owens shear (BMORS).

		Meat temperature (MT)^2^			*P* value			
Parameter	Woody Breast Category (WB)^1^	HOT	AMBIENT	COLD	WB	MT	WB x MT	Pooled SEM
BMORSF (N)^3^	NORM	7.84^d^	10.46abc	11.35^ab^	0.1419	<0.0001	0.0800	0.29
	SEV	8.54^cd^	11.61^b^	13.15^a^				
BMORSE (N.mm)^3^	NORM	97.21^e^	129.20^cd^	149.41^ab^	0.1122	<0.0001	0.0844	3.60
	SEV	106.08^de^	144.76^bc^	170.98^a^				
PC-BMORS^3^	NORM	5.00^e^	5.98^d^	7.10^bc^	<0.0001	<0.0001	0.6910	0.16
	SEV	6.39^cd^	7.31^b^	8.77^a^				

1 NORM = normal, fillets were soft and flexible throughout; SEV= severe, fillets were extremely hard and rigid throughout with limited or flexibility from cranial to caudal region.

2 Texture analysis was conducted on 3 different post-cooking meat temperature of HOT (68°C), AMBIENT (22°C), and COLD (4°C), respectively.

3 Blunt Meullenet-Owens Razor Shear (BMORS) measurements in cooked meat. BMORSF=BMORS shear force (N); BMORSE=BMORS shear energy (N.mm); PC-CBMORS=peak counts of BMORS.

a-e Means within the same parameter followed by different superscript letters differ significantly (*P* < 0.05) for each treatment group. n = 20 per mean.

### Relationship Between Meat Quality Attributes and WB Condition

Spearman's correlations between meat quality attributes of raw and cooked fillets and their WB scores (NORM/SEV analyzed as 0/2) are shown in Table 1 and Table 4. The pH, CF, and CE measurements were highly correlated with WB scores (r_s_ = 0.81, 0.92, 0.76, 0.78, and 0.78, respectively; *P* < 0.01). Color parameters (L*, a* and b*) were moderately correlated to WB scores (r_s_ = 0.42, 0.35 and 0.40, respectively). Shear values (BMORSF, BMORSE and PC-BMORS) in raw meat state were positively correlated to WB scores (*P* < 0.01) with r_s_ values equal to 0.37, 0.41 and 0.49, respectively. In cooked meat state, there were no significant Spearman correlations (*P* > 0.05) between BMORSF and BMORSE measurements and WB scores regardless of post-cooking meat temperature (HOT, AMBIENT, or COLD). However, PC-BMORS was moderately correlated (*P* < 0.01) to WB scores regardless of post-cooking meat temperature (HOT r_s_ = 0.52; AMBIENT r_s_ = 0.51; COLD r_s_ = 0.49).

**Table 4:** Pearson correlation coefficients (r) between cooked fillet shear values and cooking performance measurements and Spearman's correlation coefficients (r_s_) between shear values and WB scores.

		Cooking performance					
Shear value	Post-cooking meat temperature^1^	Total water loss	Cook loss	Moisture loss HOT^2^	Moisture loss AMBIENT^3^	Moisture loss COLD^4^	Correlations to WB scores
BMORSF	HOT	0.44⁎⁎	0.42⁎⁎	0.47⁎⁎	-0.01	-0.12	0.11
	AMBIENT	0.40⁎⁎	0.40⁎⁎	0.35⁎	0.04	-0.08	0.07
	COLD	0.56⁎⁎⁎	0.56⁎⁎⁎	0.42⁎⁎	0.18	-0.29	0.21
BMORSE	HOT	0.44⁎⁎	0.44⁎⁎	0.45⁎⁎	-0.01	-0.13	0.12
	AMBIENT	0.47⁎⁎	0.48⁎⁎	0.37⁎	0.05	-0.16	0.13
	COLD	0.58⁎⁎	0.60⁎⁎	0.43⁎⁎	0.14	-0.32*	0.22
PC-BMORS	HOT	0.29	0.21	0.36⁎	0.30	-0.11	0.52⁎⁎⁎
	AMBIENT	0.29	0.21	0.39⁎	0.26	-0.13	0.51⁎⁎⁎
	COLD	0.35⁎	0.29	0.41⁎⁎	0.25	-0.21	0.49⁎⁎⁎

1 Texture analysis was conducted at 3 different post-cooking meat temperature of HOT (68°C), AMB (23°C) and COLD (4°C), respectively.

2 HOT = moisture loss from 76°C to 68°C. ^3^ AMBIENT = moisture loss from 68°C to 22°C. ^4^ COLD = moisture loss from 22°C to 4°C.

⁎⁎⁎ *P* < 0.001; ^⁎⁎^*P* < 0.01; *P < 0.05.

Spearman's correlations between cooking time, water/moisture loss and WB scores are shown in Table 2. The length of cooking time was negatively correlated with WB scores (r_s_ = -0.53, *P* < 0.001), whereas cook loss and total water loss levels were positively correlated with WB scores (r_s_ = 0.61 and 0.73, respectively; *P* < 0.001). With exception to COLD treatment, WB scores were also correlated with moisture loss (HOT r_s_ = 0.63, AMBIENT r_s_ = 0.71; *P* < 0.001).

Pearson correlations between shear values of BMORS in cooked fillets and cooking performance (water loss properties) within each post-cooking meat temperature (HOT, AMBIENT, COLD) are shown in Table 4. BMORSF (HOT, r = 0.44; AMBIENT, r = 0.40; COLD, r = 0.56) and BMORSE (HOT, r = 0.44; AMBIENT, r = 0.47; COLD, r = 0.58) were positively correlated (*P* < 0.01) with total water loss. However, only PC-BMORS was correlated (*P* < 0.05) to total water loss in COLD (r = 0.35). Similarly, BMORSF (HOT, r = 0.42; AMBIENT, r = 0.40; COLD, r = 0.56) and BMORSE (HOT r = 0.44; AMBIENT, r = 0.48; COLD, r = 0.60) were positively correlated (*P* < 0.01) to cook loss. There were no significant Pearson correlations (*P* > 0.05) between PC-BMORS and cook loss regardless of post-cooking meat temperature (HOT, r = 0.21; AMBIENT, r = 0.21; COLD, r = 0.29). For moisture properties after cooking, BMORSF (HOT, r = 0.47; AMBIENT, r = 0.35; COLD, r = 0.42), BMORSE (HOT, r = 0.45; AMBIENT, r = 0.37; COLD, r = 0.43), PC-BMORS (HOT, r = 0.36; AMBIENT, r = 0.39; COLD, r = 0.41) were correlated with (*P* < 0.05) moisture loss (HOT) regardless of post-cooking meat temperature. There were no significant Pearson correlations (*P* > 0.05) between shear values of BMORS and moisture loss (AMBIENT), moisture loss (COLD), except the correlation (r = -0.32, *P* < 0.05) between BMORSE (COLD) and moisture loss (COLD).

Pearson correlations among cooking performance measurements are shown in Table 5. Total water loss was positively correlated (*P* < 0.001) to cook loss (r = 0.97), moisture loss (HOT) (r = 0.64) and moisture loss (AMBIENT) (r = 0.62); however, it was negatively correlated (*P* < 0.01) to moisture loss (COLD) (r = -0.44). Cook loss was positively correlated to moisture loss (HOT) (r = 0.46, *P* < 0.01), moisture loss (AMBIENT) (r = 0.51, *P* < 0.001) and was negatively correlated to moisture loss (COLD) (r = -0.43, *P* < 0.05). Moisture loss (HOT) was positively correlated to moisture loss (AMBIENT) (r = 0.37, *P* < 0.05), while moisture loss (COLD) was negatively correlated to moisture loss (HOT) (r = -0.33, *P* < 0.05) and moisture loss (AMBIENT) (r = -0.56, *P* < 0.001).

**Table 5:** Pearson correlation coefficients (r) between cooking performance measurements.

	Total water loss	Cook loss	Moisture loss HOT	Moisture loss AMBIENT	Moisture loss COLD
Total water loss	1.00				
Cook loss	0.97⁎⁎⁎	1.00			
Moisture loss HOT^1^	0.64⁎⁎⁎	0.46⁎⁎	1.00		
Moisture loss AMBIENT^2^	0.62⁎⁎⁎	0.51⁎⁎⁎	0.37⁎	1.00	
Moisture loss COLD^3^	-0.44⁎⁎	-0.43⁎⁎	-0.33⁎	-0.56⁎⁎⁎	1.00

1 HOT = moisture loss from 76°C TO 68°C.

2 AMBIENT = moisture loss from 68°C to 23°C.

3 COLD = moisture loss from 23°C to 4°C.

⁎⁎⁎ *P* < 0.001; ^⁎⁎^*P* < 0.01; *P < 0.05.

## DISCUSSION

Meat quality attributes in raw breast meat state affected by WB condition (Table 1) are similar to previous studies. SEV fillets had greater pH value (Mudalal et al., 2015; Kuttappan et al., 2017; Bowker et al., 2018; Brambila et al., 2018; Zhuang and Bowker, 2018; Baldi et al., 2019) compared to NORM fillets. Color measurements (CIE L*a*b*) on dorsal side of fillets were not fully consistent with recent published literature. In this experiment, SEV fillets exhibited greater L*, a*, and b* values than NOR fillets; however, Trocino et al. (2015) and Brambila et al. (2017) reported no significant differences in CIE L*a*b* color values between WB and unaffected breast fillets. Nevertheless, some studies reported greater L* (Baldi et al., 2019), a* (Chatterjee et al., 2016; Zhuang and Bowker, 2018), b* (Mudalal et al., 2015; Baldi et al., 2019) values for fillets severely affected by WB myopathy compared to normal samples. Differences in color measurements between WB groups reported in the literature and in the current study may be due to differences in bird age/strain, processing methodology, storage time (6 h, 18 h, 24 h, d 1−d 5), and/or color testing equipment. Previous studies have consistently reported that CF values increased as WB severity increased (Mudalal et al., 2015; Soglia et al., 2017; Sun et al., 2018; Pang et al., 2020c) regardless of sample preparation (intact fillet or portioned muscle tissue), fillet region, compression setting (% of fillet height or certain compression difference), storage time (d 0 to d 8), meat state (raw/cooked, fresh/frozen-thawed). The differences of texture characteristics (BMORSF and BMORSE) between NORM and SEV raw fillets observed in this study agreed with Bowker and Zhuang. (2019).

During cooking (5−40 min), meat temperature of SEV fillets cooked from 4°C to a final center temperature of 76°C remained higher compared to NORM fillets. Interestingly, although SEV fillets were slightly heavier and thicker than NORM fillets, they required less cooking time to reach the terminal point than NORM meat. Regardless of these slight differences in dimensional measurements between NORM and SEV fillets, this result is consistent with that recently reported by Caldas-Cueva et al. (2021a), who found that the cooking time to the terminal point was lower for patties made from WB meat compared to those produced using normal breast meat. To minimize the effects of heterogeneity among raw samples, those authors weighed a fixed amount of each ground breast meat, which was formed into a uniform and consistent circular-shaped patty. The differences in meat temperature during thermal processing and the length of cooking time between SEV and NORM fillets may be associated with their differences in physicochemical and histological properties (Sihvo et al., 2014; Soglia et al., 2016a,b). For instance, Caldas-Cueva et al. (2021a) hypothesized that the reduced cooking time observed in WB patties could be attributed to the fact that WB fillets typically have a greater moisture content compared to normal breast meat; it means that WB patties containing higher water content could experience alterations in their thermophysical properties such as the increase of thermal conductivity and diffusivity, which could reduce the cooking time of these poultry products. Thus, special attention should be given to the cooking process of chicken breast fillets because WB condition could impact food safety and quality. NORM meat could be potentially undercooked if a SEV WB meat product was chosen in a random sample for temperature monitoring. Furthermore, Pang et al. (2020b) demonstrated that 3 distinct water components (hydration water, intra-myofibrillar water and extra-myofibrillar water) measured by time-domain nuclear magnetic resonance (TD-NMR) were greater in intact breast fillets with WB condition compared to normal fillets, which may have an influence on functional properties such as water-binding ability. The findings in that study indicate the mobility of water properties in WB is greater than NORM fillets, which may cause the reduction of WHC (greater cook loss) in WB. In this study, SEV fillets have greater cook loss and/or total water loss compared to NORM fillets which was consistent with published data (Mudalal et al., 2015; Tasoniero et al., 2016; Tijare et al., 2016; Sanchez Brambila et al., 2018). In addition, strong correlations between cook loss/total water loss (r = 0.61 and 0.73 respectively, Table 2) and WB scores confirmed the poor WHC of WB fillets during thermal processing. Our data further demonstrate greater moisture loss (HOT, AMBIENT) in SEV fillets during cooling down process (76°C−22°C) than NORM fillets. The results of moisture loss levels suggest the poor ability of WB meat to retain water after cooking compared to unaffected normal meat.

Shear values of BMORS were conducted on cranial region of NORM and SEV fillets at varying post-cooking meat temperatures (HOT, AMBIENT, COLD). Results from BMORS test indicated that SEV and NORM fillets had similar BMORSF and BMORSE values (*P* > 0.05). Previous research have shown mixed results. Researchers have that reported that WB had greater shear force and energy regardless of shearing methodologies (Warner-Bratzler shear, MORS/BMORS, Allo-Kramer method), bird age (small vs big) or sample preparation (intact breast fillet vs portioned muscle) (Tasoniero et al., 2016; Chatterjee et al., 2016; Petracci et al., 2019; Mallmann et al., 2020). Other studies have reported greater values of texture profile analysis hardness in non-marinated or marinated cooked WB fillets/products with higher cook-loss percentages compared to unaffected fillets (Soglia et al., 2016b; Caldas-Cueva et al., 2020). However, other researchers did not observe differences (Mudalal et al., 2015; Tijare et al., 2016; Cai et al., 2018; Dalgaard et al., 2018); in fact, Byron et al. (2020) reported NORM fillets had greater shear force in different fillet regions (cranial, middle, caudal) than SEV fillets on d 0 cooking (processing day). The PC-BMORS in cooked meat increased significantly (*P* < 0.05) as WB severity increased, which was consistent with previous studies (Sun et al., 2016; Bowker and Zhuang, 2019). In addition, only PC-BMORS correlated to WB scores in all HOT, AMBIENT, and COLD treatments (Table 4). The results of this study along with other studies suggest that shear force or shear energy of cooked breast fillets between WB categories can be inconsistent, which might be related to multiple factors such as shearing methodology, water holding capacity, bird age, deboning time, sample size, fillet region/location, cooking methods, etc. However, PC-BMORS objectively distinguished fillets with WB condition in this study and was consistent with the results from previous studies (Sun et al., 2016) suggesting that PC-BMORS in shear analysis could be a useful as a texture indicator feature for WB classification.

In the current study, the data further indicated that shear values increased as post-cooking meat temperature decreased (HOT < AMBIENT < COLD). Solo (2016) reported that higher juiciness, flavor, and tenderness sensory scores were associated with hot meat samples compared to cold meat samples. The results in this study indicated that moisture loss during high temperatures (HOT) was moderately correlated (*P* < 0.05) to shear properties whereas moisture loss from HOT to AMBIENT or COLD temperatures were not correlated (*P* > 0.05) suggesting that moisture loss in the early period after cooking may affect shear values to a greater extent. Shear values showed an increasing trend as meat temperature decreased due to sample cooling which impacts moisture, but potentially also collagen and fat properties that may affect the hardness of the meat. Future work is needed to determine other contributing factors to increasing hardness of poultry meat as cooked product temperature decreases. It is important to note that the meat temperature could be hard to maintain HOT when assessing many samples in short time and this could lead to inconsistent results. If samples were held in controlled manner at high temperature (e.g., using holding oven), it would also be important to control moisture loss during holding.

## CONCLUSION

There was evidence of the poor meat quality associated with raw fillets severely affected by WB condition, which exhibited greater values of objective color parameters, pH, and instrumental texture measurements compared to normal fillets. During thermal processing, meat temperature of SEV fillets was higher than NORM fillets from 5 min to 40 min and reached final endpoint temperature of 76°C faster than NORM fillets. Poor cooking performance and textural attributes were attributed to the WB condition. These data also suggest that temperature at which fillets are sheared (post-cooking meat temperature) plays an important role in instrumental texture results and thus, it is important to maintain constant conditions during analysis and compare data across studies using taking into account the hearing procedures used.

## ACKNOWLEDGMENTS

The authors are appreciative for the support of the 10.13039/100007755University of Arkansas Division of Agriculture (Fayetteville, AR) and the 10.13039/100007756University of Arkansas Processing Plant for providing technical support during data collection. The authors would like to acknowledge the funding from 10.13039/501100009006Chuzhou University (Anhui, China) and the program (JQLAB-KF-201901) for visiting scholar support. Meanwhile, the authors also appreciate for the São Paulo Research Foundation (FAPESP) for the support provided (2019/09707-6).

## Disclosures

There is no conflict of interest.
